# Clinical outcomes from robotic transabdominal preperitoneal inguinal hernia repair in patients under and over 70 years old: a single institution retrospective cohort study with a comprehensive systematic review on behalf of TROGSS - The Robotic Global Surgical Society

**DOI:** 10.1007/s40520-024-02890-9

**Published:** 2024-12-24

**Authors:** Yeisson Rivero-Moreno, Aman Goyal, Samantha Redden-Chirinos, Halil Bulut, Rebeca Dominguez-Profeta, Pujita Munnangi, Jason Shenoi, Paulamy Ganguly, Pierre Blanc, Khalid Alkadam, Sjaak Pouwels, Safwan Taha, Beniamino Pascotto, Juan Santiago Azagra, Wah Yang, Andrea Garcia, Kathia Dayana Morfin-Meza, Clotilde Fuentes-Orozco, Alejandro González-Ojeda, Luis Osvaldo Suárez-Carreón, Luigi Marano, Adel Abou-Mrad, Rodolfo J. Oviedo

**Affiliations:** 1 Department of Surgery, Montefiore Medical Center, New York, USA; 2 Universidad de Oriente, Núcleo Anzoátegui, Venezuela; 3 Adesh Institute of Medical Sciences and Research, Bathinda, Punjab India; 4 Istanbul University Cerrahpasa, Istanbul, Turkey; 5 Texas A&M University School of Medicine, 1020 Holcombe Blvd, Houston, TX USA; 6 School of Engineering Medicine, Texas A&M University, 1020 Holcombe Blvd, Houston, TX USA; 7 Centre mutualiste de l’obésité, Clinique Chirurgicale Mutualiste de, Saint Etienne, France; 8 Ad Diriyah Hospital, Riyadh, Saudi Arabia; 9 Department of Surgery, Marien Hospital Herne, University Hospital of Ruhr University Bochum, Herne, NRW Germany; 10 Department of Intensive Care Medicine, Elisabeth-Tweesteden Hospital, Tilburg, the Netherlands; 11 The Metabolic and Bariatric Surgery Centre (COEMBS), Mediclinic Hospital Airport Road, Abu Dhabi, United Arab Emirates; 12 Centre Hospitalier de Luxembourg, Luxembourg City, Luxembourg; 13 The First Affiliated Hospital of Jinan University, Guangzhou, China; 14 Unidad, De Investigación Biomédica 02, Hospital De Especialidades Del Centro Médico Nacional De Occidente, Guadalajara, Mexico; 15 Facultad De Medicina De La Universidad De Colima, Colima, Mexico; 16 UMAE Hospital de Especialidades del Centro Medico Nacional de Occidente, Guadalajara, Mexico; 17 University of Guadalajara, Guadalajara, Mexico; 18 Department of Medicine, Surgery, and Neurosciences, University of Siena, Viale Bracci 3, 53100 Siena, Italy; 19 Department of Medicine, Academy of Applied Medical and Social Sciences-AMiSNS, Akademia Medycznych I Spolecznych Nauk Stosowanych, 2 Lotnicza Street, 82-300 Elbląg, Poland; 20 Department of General Surgery and Surgical Oncology, “Saint Wojciech” Hospital, “Nicolaus Copernicus” Health Center, Jana Pawła II 50, 80-462 Gdańsk, Poland; 21 Centre Hospitalier Régional et Universitaire d’Orleans, Orléans, France; 22 Department of Surgery, Nacogdoches Medical Center, Nacogdoches, TX USA; 23 University of Houston Tilman J. Fertitta Family College of Medicine, Houston, TX USA; 24 Sam Houston State University College of Osteopathic Medicine, Conroe, TX USA

**Keywords:** Robotic surgery, Inguinal hernia, Postoperative complications, Age groups

## Abstract

**Aim:**

This study aimed to assess and compare outcomes of robotic inguinal hernia repair (RIHR) in patients under and over 70 years old, performed by a fellowship-trained robotic surgeon at a single institution.

**Methods:**

A retrospective analysis of patients undergoing robotic primary transabdominal preperitoneal inguinal hernia repair between 2020 and 2022 was conducted. Patients were categorized into two age groups: those under 70 years and 70 years and older. Data were collected through chart reviews with a mean follow-up of 30 days. Concurrently, a systematic review (SR) of relevant high-level literature was carried out.

**Results:**

Among the 37 patients studied, 75.7% (n = 28) were male, with a mean age of 64.8 years. Demographic features did not significantly differ based on age groups. Patients > 70 years had a higher incidence of reported complications (52.3% vs. 87.5%, p < 0.461). There were no differences in operative time or length of stay between the groups. In the SR, only 23.7% (n = 9) of studies provided age-related conclusions. Three studies identified age over 70 as a risk factor for postoperative complications, while two studies suggested that RIHR is feasible and safe in patients aged 80 years and older.

**Conclusion:**

Patients over 70 years old demonstrated a higher incidence of complications compared to younger patients. However, current literature indicates that the robotic approach may offer a safe and minimally invasive option for inguinal hernia repair in both younger and older adults.

## Introduction

Inguinal hernias occur in 3–6% of women and 27–43% of men over their lifetimes. Almost usually symptomatic, inguinal hernias can only be treated surgically. A small percentage of patients experience no symptoms at all, yet even in this group, 70% of cases require surgery within five years of starting treatment. With more than 20 million cases each year, inguinal hernia repairs are among the most common surgical procedures carried out globally [1].

Minimally invasive methods have replaced open procedures in the surgical treatment of inguinal hernias. For primary unilateral inguinal hernias, current guidelines favor laparoscopy due to its ability to reduce postoperative pain, wound infection, and recovery time compared to open surgery [2].

One of the most recent advances in minimally invasive procedures is the use of robotic platforms. Notably, the da Vinci system from Intuitive Surgical offers an enhanced range of motion, a sturdy platform, and a magnified three-dimensional (3D) view [3]. Thus, surgeons could be able to overcome the technical challenges associated with laparoscopic hernia repair, perhaps leading to improved clinical results [4].

Numerous studies have detailed how surgeons successfully switched from a laparoscopic to a robotic technique, mastering the learning curve and proving the approach’s viability and safety profile [5–7]. The laparoscopic technique for mesh implantation in the preperitoneal area was first established in the early 1990s. The laparoscopic inguinal hernia repair has not gained popularity among surgeons, and the technique’s growth has stagnated for years despite these well-established benefits and published guidelines. This could be due to the steep learning curve and need for advanced laparoscopic techniques. Due to its potential for improved dexterity, safety, and cosmetic results, robotic surgery (RS) has grown in popularity. It offers advantages over laparoscopy in terms of ergonomics, wristed articulating devices, improved three-dimensional imaging, and simplicity of intracorporeal suturing [8].

Inguinal hernia repair using the transabdominal preperitoneal (TAPP) technique is a procedure that can benefit from robotic assistance [9]. Several studies had already used RS to show the viability and advantages of this strategy [10–12]. The number of older adults is at an all-time high globally. Although older patients may be able to wait patiently for moderately symptomatic inguinal hernias, elderly people now lead more active lifestyles and want a quicker recovery after surgery so they may resume their regular activities [13]. Given that some studies indicate a higher likelihood of perioperative problems in surgical candidates for inguinal hernia repair, advanced age may be a significant comorbidity [14–16]. Research comparing laparoscopic and open inguinal hernia repair in elderly adults has confirmed the safety and efficacy of both techniques in this patient group [13]. The purpose of this study was to present data that would help explain how age affects the clinical results of robotic inguinal hernia repairs in patients who are 70 years of age or older. Although the robotic TAPP inguinal hernia repair has been covered in earlier papers, a thorough systematic review has not yet been conducted to address the pertinent question of how aging affects clinical results in this setting.

## Materials and methodology

### Study design

A retrospective analysis of a prospectively maintained database was conducted under Institutional Review Board (IRB) approval (Approval Number: #PRO00031398) at a single academic institution in Houston, Texas, United States. The study included patients who underwent robotic TAPP inguinal hernia repair between November 2020 and November 2022. All procedures were performed by a fellowship-trained and board-certified minimally invasive and robotic surgeon within a general surgery residency program.

### Study population

Adult patients (≥ 18 years) who underwent robotic TAPP inguinal hernia repair during the study period were included. Patients were stratified into two age groups for analysis: those under 70 years and those aged 70 years and older. Demographic and clinical variables, including age, sex, body mass index (BMI), American Society of Anesthesiologists (ASA) classification, and comorbidities, were recorded.

Exclusion criteria included patients who underwent concurrent procedures, lacked sufficient follow-up data, or had incomplete records in the database.

### Data collection

Primary outcomes included operative variables and clinical outcomes. Data were collected through chart reviews and included:

- Demographic variables: age, sex, body mass index (BMI), American Society of Anesthesiologists (ASA) classification, and comorbidities
- Operative parameters: mean operating time (OT) and length of hospital stay (LOS).
- Clinical outcomes: incidence of surgical site infection (SSI), seroma formation, urinary retention, hernia recurrence, chronic pain lasting > 30 days, and reoperation rates.

### Statistical analysis

Quantitative variables were expressed as median and standard deviation (SD), and qualitative variables as proportions. The Chi-square and Fisher’s exact were used for categorical variables, and the student’s t-test was used for quantitative variables; the Monte Carlo simulation method was used due to the low sample size. The statistical software IBM SPSS Statistics for Windows, Version 29.0.2.0 (Armonk, NY: IBM Corp.) [17], was used to conduct statistical tests to assess for statistical significance (p < 0.05).

### Systematic review

A systematic review was conducted alongside the retrospective analysis to identify age-group-specific clinical outcomes and gather the most pertinent high-level literature on robotic inguinal hernia repair (RIHR). The review followed the Preferred Reporting Items for Systematic Review and Meta-Analysis Protocols (PRISMA-P 2020) guidelines [18].

### Search strategy

A comprehensive search strategy was designed to identify studies reporting age-specific clinical outcomes following robotic inguinal hernia repair (RIHR). The last search, conducted on December 15, 2023, encompassed the PubMed, Scopus, Web of Science, and ScienceDirect databases using the following search terms: “robotic surgical procedures,” “inguinal hernia,” and “age”. Detailed search strategies are provided in Table 1.

**Table 1:** Search terms for the meta-analysis in PubMed, Scopus, Web of Science, and Science direct databases

Database	Search terms
PubMed (n = 81)	("age"[All Fields]) **AND** ("hernia, inguinal"[MeSH Terms] OR ("hernias inguinal"[Title/Abstract] OR "Inguinal Hernias"[Title/Abstract] OR "Inguinal Hernia"[Title/Abstract] OR "inguinal hernia indirect"[Title/Abstract] OR "hernia indirect inguinal"[Title/Abstract] OR "hernias indirect inguinal"[Title/Abstract] OR "Indirect Inguinal Hernia"[Title/Abstract] OR "Indirect Inguinal Hernias"[Title/Abstract] OR "inguinal hernias indirect"[Title/Abstract] OR "inguinal hernia direct"[Title/Abstract] OR "Direct Inguinal Hernia"[Title/Abstract] OR "Direct Inguinal Hernias"[Title/Abstract] OR "hernia direct inguinal"[Title/Abstract] OR "hernias direct inguinal"[Title/Abstract] OR "inguinal hernias direct"[Title/Abstract])) **AND** ("Robotic Surgical Procedures"[MeSH Terms] OR ("Robotic Surgical Procedures"[Title/Abstract] OR "Robotic Surgical Procedure"[Title/Abstract] OR "Robot Surgery"[Title/Abstract] OR "Robot Surgeries"[Title/Abstract] OR "Robotic Surgery"[Title/Abstract] OR "Robotic Surgeries"[Title/Abstract] OR "robot assisted surgery"[Title/Abstract] OR "robot assisted surgery"[Title/Abstract] OR "robot assisted surgeries"[Title/Abstract] OR "robot assisted surgeries"[Title/Abstract] OR "robot enhanced procedures"[Title/Abstract] OR "robot enhanced procedures"[Title/Abstract] OR "robotic assisted surgery"[Title/Abstract] OR "robotic assisted surgery"[Title/Abstract] OR "robotic assisted surgeries"[Title/Abstract] OR "robotic assisted surgeries"[Title/Abstract] OR "robot enhanced surgery"[Title/Abstract] OR "robot enhanced surgery"[Title/Abstract]))
Scopus (n = 116)	( TITLE-ABS-KEY ( age)) **AND** ( TITLE-ABS-KEY ( "Inguinal Hernias" OR "Inguinal Hernia" OR "Indirect Inguinal Hernia " OR "Indirect Inguinal Hernias " OR "Direct Inguinal Hernia " OR "Direct Inguinal Hernias ")) **AND** ( TITLE-ABS-KEY ( "Robotic Surgical Procedures" OR "Robotic Surgical Procedure" OR "Robot Surgery" OR "Robot Surgeries" OR "Robotic Surgery" OR "Robotic Surgeries" OR "Robot-Assisted Surgery" OR "Robot Assisted Surgery" OR "Robot-Assisted Surgeries" OR "Robot Assisted Surgeries " OR "Robot-Enhanced Procedures" OR "Robot-Enhanced Procedure" OR "Robot Enhanced Procedure" OR "Robot Enhanced Procedures" OR "Robotic-Assisted Surgery" OR "Robotic Assisted Surgery" OR "Robotic-Assisted Surgeries" OR "Robotic Assisted Surgeries" OR "Robot-Enhanced Surgery" OR "Robot Enhanced Surgery" OR "Robot-Enhanced Surgeries" OR "Robot Enhanced Surgeries " OR "Robotic-Enhanced Surgery" OR "Robotic Enhanced Surgery" OR "Robotic-Enhanced Surgeries" OR "Robotic Enhanced Surgeries "))
Web of Science (n = 84)	TS = (age) **AND** TS = (Robotic Surgical Procedures OR Robotic Surgical Procedure OR Robot Surgery OR Robot Surgeries OR Robotic Surgery OR Robotic Surgeries OR Robot-Assisted Surgery OR Robot Assisted Surgery OR Robot-Assisted Surgeries OR Robot Assisted Surgeries OR Robot-Enhanced Procedures OR Robot-Enhanced Procedure OR Robot Enhanced Procedure OR Robot Enhanced Procedures OR Robotic-Assisted Surgery OR Robotic Assisted Surgery OR Robotic-Assisted Surgeries OR Robotic Assisted Surgeries OR Robot-Enhanced Surgery OR Robot Enhanced Surgery OR Robot-Enhanced Surgeries OR Robot Enhanced Surgeries OR Robotic-Enhanced Surgery OR Robotic Enhanced Surgery OR Robotic-Enhanced Surgeries OR Robotic Enhanced Surgeries) **AND** TS = (Hernias, Inguinal OR Inguinal Hernias OR Inguinal Hernia OR Inguinal Hernia, Indirect OR Hernia, Indirect Inguinal OR Hernias, Indirect Inguinal OR Indirect Inguinal Hernia OR Indirect Inguinal Hernias OR Inguinal Hernias, Indirect OR Inguinal Hernia, Direct OR Direct Inguinal Hernia OR Direct Inguinal Hernias OR Hernia, Direct Inguinal OR Hernias, Direct Inguinal OR Inguinal Hernias, Direct)
Science Direct (n = 11)	Title, abstract, keywords: (robotic surgery OR robot surgical procedure) **AND** (inguinal hernia OR indirect inguinal hernia OR direct inguinal hernia) **AND** (age)

### Study selection and eligibility criteria

Two independent reviewers screened titles and abstracts for relevance, followed by a full-text review of potentially eligible studies. Disagreements were resolved through discussion or consultation with a third reviewer. Inclusion criteria were as follows:

- Studies reporting on patients undergoing RIHR.
- Studies describing age-stratified clinical outcomes.
- Cohort, case–control, cross-sectional studies, or randomized controlled trials.
- The language and publication date were both unrestricted.

Exclusion criteria included:

- Studies involving hernia repair combined with other surgical procedures.
- Studies with overlapping populations derived from the same database.

### Data extraction

Data were extracted independently by two reviewers and included:

- Study characteristics: authors, year of publication, country, study design, and sample size.
- Patient demographics: age, sex, and proportion of bilateral hernia repairs.
- Outcomes: operative time, complication rates, and hernia recurrence.

### Ethical approval

The study adhered to the ethical principles outlined in the Declaration of Helsinki. Institutional Review Board approval (Approval Number: #PRO00031398) was obtained prior to data collection and analysis.

## Results

Data from 37 patients who underwent robotic TAPP inguinal hernia repair was analyzed. Overall, 75.7% (n = 28) of the patients were male, with a mean age of 64.8. The average BMI was 25.62. ASA classification II was the most common group, reported in 56.8% (n = 21) of the patients. History of abdominal/pelvic/urologic surgery was present in 27.02% (n = 10) of the patients, and hypertension was the most common comorbidity (35.13%). The detailed demographic features, according to age group, are shown in Table 2. There were no statistically significant differences in the demographic features of the patients in this study based on the age group.

**Table 2:** Demographic Breakdown of Patients by Age Group

Features ^a^	< 70 years (n = 21)	≥ 70 years (n = 16)	P value
Age	54.24 ± 10.76	75.44 ± 3.82	< 0.001 *
BMI (kg/m^2^**)**	27.25 ± 7.15	23.99 ± 2.90	0.095^†^
*Sex*			
Male	16 (57.1)	12 (42.9)	0.615^†^
Female	5 (55.6)	3 (44.4)	
*ASA Class n (%)*			
Class I	1 (4.8)	0	0.310^‡^
Class II	13 (61.9)	8 (50.0)	0.190^†^
Class III	7 (33.3)	6 (37.5)	0.760^†^
Class IV	0	2 (12.5)	0.152 ^‡^
*Comorbidities & risk factors n (%) *^**b**^			
History of abdominal/pelvic/urologic surgery	5 (23.81%)	5 (31.25%)	0.8014^†^
Coronary artery disease	4 (19.05%)	5 (31.25%)	0.5582^†^
Hypertension	6 (28.57%)	7 (43.75%)	0.4458^†^
GERD	4 (19.05%)	6 (37.5%)	0.3142^†^
Cancer	5 (23.81%)	6 (37.5%)	0.5011^†^
Obesity	3 (14.29%)	1 (6.25%)	0.8577^†^
Diabetes mellitus	0 (0%)	1 (6.25%)	0.8639^†^
Liver disease	3 (14.29%)	0 (0%)	0.5992^†^
Kidney disease	1 (4.76%)	3 (18.75%)	0.37^†^

ASA class: American Society of Anesthesiologists Classification, *BMI* Body Mass Index, *GERD* Gastroesophageal Redux Disease.

^a^Continuous data are shown as the mean ± standard deviation and categorical data as number (%)

^b^One patient had more than one comorbidity. Percentages were calculated from the total of patients of the age group.

*T-Student

^†^Chi-square

^‡^Fisher exact test

Bold values are statistically significant

There were no significant differences in surgical outcomes and complication rates between the two age groups, as shown in Table 3.

**Table 3:** Robotic Inguinal Hernia Repair Surgery Features and Outcomes by Age

Features	Total (n = 37)	< 70 years (n = 21)	≥ 70 years (n = 16)	P value
*Surgery outcomes*^a^				
Mean Operating Time (min)	97.76	93.90	102.81	0.372*
Length of Hospital Stay (hr)	193.65	173.24	220.44	0.090*
*Surgical complications*				
Seroma Formation	12 (32.4)	7 (33.3)	5 (31.3)	0.528^†^
Chronic Pain (> 30 days)	6 (16.2)	2 (9.5)	4 (25)	0. 394^‡^
Reoperation	3 (8.1)	1 (4.8)	2 (12.5)	0. 556^‡^
Hernia Recurrence	3 (8.1)	1 (4.8)	2 (12.5)	0. 556^‡^
Urinary Retention	1 (2.7)	0	1 (6.3)	0. 314^‡^
Total number of complications reported	25 (67.5)	11 (52.3)	14 (87.5)	< 0.461^†^

^a^Continuous data are shown as the mean ± standard deviation and categorical data as number (%)

*T-Student

^†^Chi-square

^‡^Fisher exact test

Bold values are statistically significant

### Systematic review

**Fig. 1:**
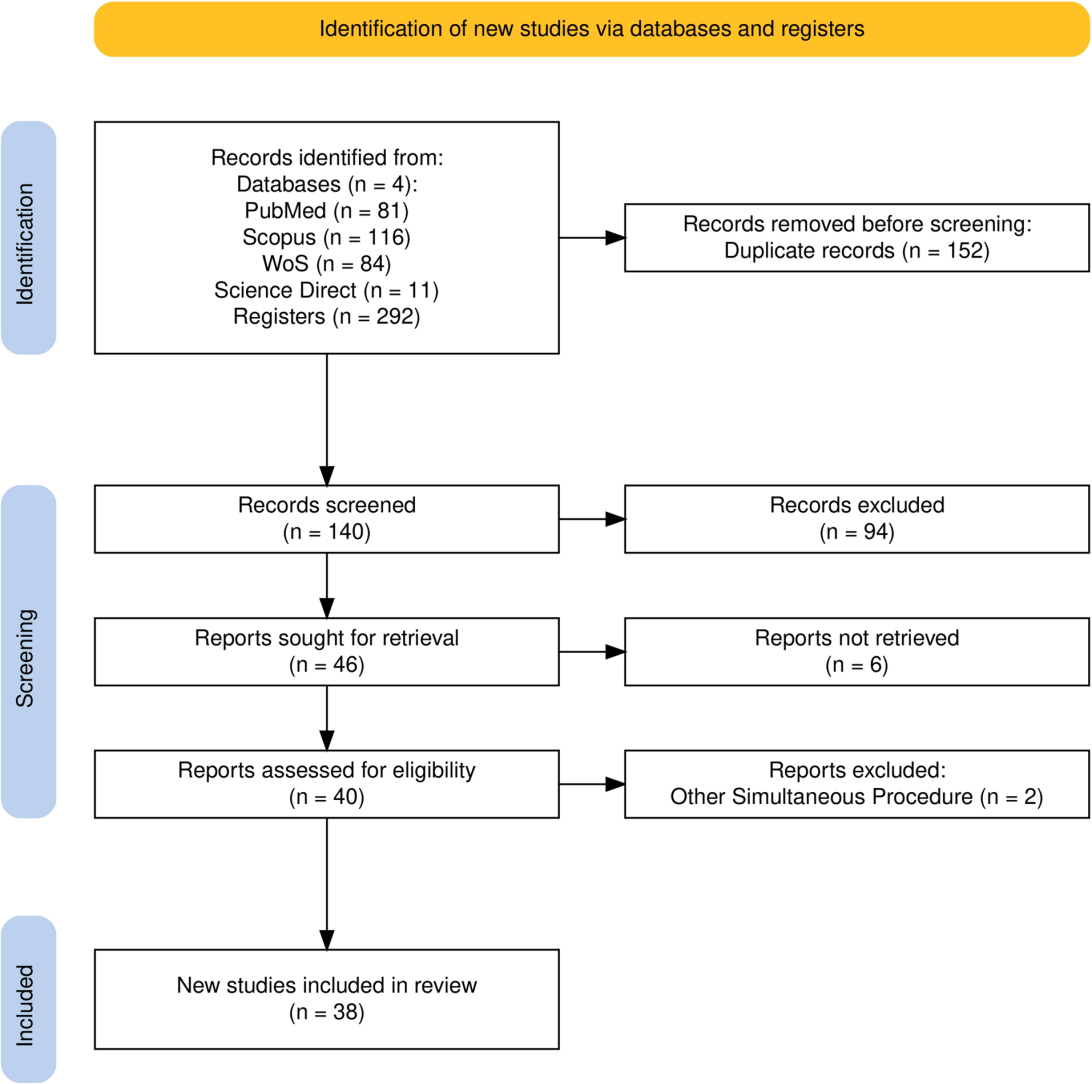
Search outputs based on PRISMA guidelines [18]

In the first literature search, we found 292 records from four distinct databases, of which 152 were duplicates. In the end, we extracted data from 38 English-language papers after completing title and abstract screening, full-text review, and inclusion and exclusion criteria in advance. The specifics of the article selection procedure are displayed. Figure 1‘s PRISMA flowchart was organized using the Haddaway et al. web application [19].

The 38 studies analyzed were published from 2011 to 2023, with most being published around 2020. These studies primarily consisted of 26 retrospective cohort studies, 10 prospective cohort studies, and 2 randomized controlled trials. The data spanned a median of three years. Most studies were conducted in the USA (68%), followed by England (8%), and a few from Brazil, South Korea, Turkey (5% each), and Australia, Germany, Italy, and Switzerland (3% each). Altogether, the studies involved 500,812 patients, with an average age of 54 and 89% being male. About 45% of the studies included a comparison group with either open or laparoscopic procedures alongside robot-assisted inguinal hernia repair (RIHR). Detailed characteristics of the studies, patient demographics, and outcomes are provided in Table 4.

**Table 4:** Summary and baseline data of the included studies

Study	Country	Type of study	Years evaluated	Number of patients (% male)	Mean age—(SD / range)	Bilateral hernia repair (%)	Type of Procedure	Evaluated Outcomes	Conclusion based on age
Jung et al. (2023) [4]	South Korea	Retrospective cohort study	2022–2023	21 (95.2)	54.1 (16.4)	19	RTAPP + synthetic mesh	Mean operation and console times, postoperative complications, LOS	No conclusion based on age
Peltrini et al. (2023) [5]	Italy	Retrospective cohort study	2015–2020	120 (88.5)	56 (13)	120	LTAPP and RTAPP	OT, complications, LOS, VAS, recurrence	No conclusion based on age
Morrell et al. (2021) [8]	Brazil	Retrospective cohort study	2016–2020	97 (90.7)	36.4 (22–71)	19	RTAPP	OT, complications, recurrence rate, LOS	No conclusion based on age
Waite et al. (2016) [9]	USA	Retrospective cohort study	2012–2014	63 (98.4)	57.5 (43–72)	6	RTAPP and LTAPP	Mean OT, recovery room time, average pain scores in recovery, average direct cost, average contribution margin	No conclusion based on age
Kakiashvili et al. (2021) [10]	USA	Prospective cohort study	2013–2015	100 (62)	56 (NR)	22	RTAPP + syntetic meh	OT, LOS, VAS, complications, recurrence rate	No conclusion based on age
Gamagami et al. (2018) [11]	USA	Retrospective cohort study	2006–2015	1254 (90.1)	56.5 (16.1)	315	RTAPP	LOS, postoperative complication, conversion, transfusion,	**Age > 65 years is a risk factor for complications within 30 days post discharge (OR = 3.33, 95% CI 1.89, 5.87; p < 0.0001)**
Engan et al. (2015) [12]	USA	Retrospective cohort study	2013	34 (88.2)	49.3 (16–80)	9	RTAPP	Mean OT, postoperative complications	No conclusion based on age
Bilezikian et al. (2021) [20]	USA	Randomized controlled trial	2015–2016	50 (77)	63 (30–87)	17	RIHR and LIHR + Syndetic mesh	Mesh insertion time, difficulty of placement	No conclusion based on age
Dominguez et al. (2016) [21]	USA	Retrospective cohort study	2014–2015	78 (91)	55.1 (15.1)	45	RTAPP + synthetic mesh	Postoperative complications	No conclusion based on age
Charles et al. (2018) [22]	USA	Retrospective cohort study	2012–2016	510 (93.3)	55 (39–57)	0	RHIR, LHIR, and OHIR	OT, adverse events, readmissions, and death, Cost, SSI	**Age was significantly different between the three groups**
Aghayeva et al. (2020) [23]	Turkey	Prospective cohort study	2016–2018	86 (93)	52.2 (16.7)	44	LTEP and RTAPP	OT, VAS scores at 24 h after surgery, Total hospital costs, Early complications, LOS, Late complications	No conclusion based on age
Aghayeva et al. (2020) [24]	Turkey	Retrospective cohort study	2016–2019	50 (96)	51.7 (16.9)	22	RTAPP	OT, LOS, estimated blood loss, VAS, Complication, Learning curve	No conclusion based on age
Proietti et al. (2021) [25]	Switzerland	Prospective cohort study	2017–2019	132 (100)	60.1 (13.7)	38	RTAPP	Recurrence, Conversion to open surgery or laparoscopy, intraoperative complication, learning curve, OT	No conclusion based on age
Iraniha et al. (2018) [26]	England	Prospective cohort study	2012–2015	82 (100)	52.86 (17–83)	77	RTAPP + synthetic meh	Docking, console, and total operating time, pain score, quality of life, postoperative complications	No conclusion based on age
Pokala et al. (2019) [27]	USA	Retrospective cohort study	2013–2017	3547 (86.5)	51 (NR)	nr	RHIR, LHIR, and OHIR	Postoperative complications, 30-day readmission, mortality, LOS, and intra-hospital opiate utilization	No conclusion based on age
Yu et al. (2020) [28]	USA	Retrospective cohort study	2017–2019	26 (100)	57.5 (11.6)	16	RTEP + primary fascial closure and pre-peritoneal mesh	OT, VAS, LOS, perioperative complications, time to return to normal activity, and the modified Medical Outcome Study score	No conclusion based on age
Florin et al. (2022) [29]	England	Prospective cohort study	2017–2018	111 (100)	18–93	39	RTAPP	OT, blood loss, conversion rates, recurrence, chronic pain	No conclusion based on age
Yoo et al. (2023) [30]	South Korea	Retrospective cohort study	2021–2022	35 (97.1)	55.03 (18.2)	16	RTAPP	OT, intra and postoperative complications,	No conclusion based on age
Huerta et al. (2019) [31]	USA	Retrospective cohort study	2005–2017	1299 (100)	56.5 (12.56)	226	RHIR, LHIR, and OHIR	OT, LOS, Recurrence, Inguinodynia, Postoperative inguinal pain, SS, Urinary retention, Ileus, Ischemic orchitis, Hematoma/seroma, Major complications (bowel perforation, aspiration pneumonia, MI, DVT)	**Age was higher in the OHIR group**
Khoraki et al. (2020) [32]	USA	Retrospective cohort study	2015–2017	183 (94.8)	49.8 (13.5)	43	RHIR, LHIR, and OHIR	OT, costs, readmissions	No conclusion based on age
Vossler et al. (2019) [33]	USA	Retrospective cohort study	2010–2015	102,241 (93)	< 65 años 70.8%	0	RHIR and LHIR	Likelihood to underwent LHIR or RIHR	No conclusion based on age
Ebeling et al. (2020) [34]	USA	Prospective cohort study	2017–2019	96 (89.6)	54.7 (15.9)	56	RTAPP	Resident competency scores, autonomy scores, operative times, complications	No conclusion based on age
Gerdes et al. (2022) [35]	Germany	Prospective cohort study	2020	58 (88)	57.5 (21–81)	7	RHIR and LHIR	OT, perioperative complications, postoperative pain	No conclusion based on age
Pereira et al. (2022) [36]	England	Retrospective cohort study	2016–2020	312 (69)	54 (16)	48	RTAPP	Postoperative complications within 30 days	**Each additional year of age was associated with a 2.1% increased risk of complications within 30 days**
Janjua et al. (2020) [37]	USA	Retrospective cohort study	2009–2015	103,183 (92.2)	58.36 (15)	5054	RHIR and OHIR	Charlson Comorbidity Category, insurance coverage, median income quartile, routine discharge disposition, Likelihood to underwent OHIR or RIHR	**In the laparoscopic and robotic groups, the patients were more frequently older than 31 years**
Tam et al. (2019) [38]	USA	Retrospective cohort study	2015–2017	335 (93)	59 (14.1)	130	RHIR	OT, complications, readmissions, follow up outcomes	No conclusion based on age
Arcerito et al. (2016) [39]	USA	Prospective cohort study	2013–2015	78 (79.5)	56 (25–96)	22	RTAPP + syntetic meh	SSI, Urinary retention, hematomas, postoperative seromas, Postoperative pain, admitted overnight, OT	**Advanced age was a factor for overnight admission in 3 patients**
Prabhu et al. (2020) [40]	USA	Randomized controlled trial	2016–2019	102 (90.2)	56.7 (13.7)	0	RHIR and LHIR	VAS, health-related quality of life, mobility, wound morbidity, cosmesis, cost, surgeon ergonomics, and multidimensional workload	No conclusion based on age
Amaral et al. (2022) [41]	Brazil	Prospective cohort study	2015–2020	19 (95)	55.2 (13.1)	8	RTAPP	30-day surgical site occurrences, SSI, hernia recurrence rates	No conclusion based on age
Tran et al. (2011) [42]	Autralia	Prospective cohort study	2010	32 (NR)	47 (NR)	NR	RTEP and LTEP	OT, number of times scope needed cleaning, time spent cleaning scope, SSI, LOS, patient satisfaction, recurrence	No conclusion based on age
Cuccurullo et al. (2020) [43]	USA	Retrospective cohort study	2016–2018	32 (84.3)	48.6 (20–67)	7	RTAPP	OT, complications, recurrence rate, LOS	No conclusion based on age
Maas et al. (2021) [44]	USA	Retrospective cohort study	2015–2018	43 (93)	56 (18–85)	12	RTAPP	Complications, OT, LOS, recurrence rates, postoperative outcomes like seroma, hematoma, SSI	**Robotic surgery is a safe option for patients on the age range of 18–85 years**
Sheldon et al. (2019) [45]	USA	Retrospective cohort study	2016–2018	173 (92.2)	38.2 (11)	46	OIHR, LIHR, and RTAPP	Initial postoperative opioid prescription, repeat opioid prescription within 3 months, postoperative NSAID and acetaminophen prescription	No conclusion based on age
Howard et al. (2023) [46]	USA	Retrospective cohort study	2020–2021	5269 (43.6)	53.8 (14.5)	NR	RHIR, LHIR, and OHIR	Postoperative complications, emergency department visits, hospital readmissions, and reoperations	No conclusion based on age
Forester et al. (2021) [47]	USA	Retrospective cohort study	2009–2019	795 (43.5)	64 (14)	NR	OIHR, LIHR, and RTAPP	LOS, VAS, time to return to normal activities, SSI, seromas, quality of life scores	No conclusion based on age
Tatarian et al. (2023) [48]	USA	Retrospective cohort study	2010–2016	280,064 (69.4)	55.3 (15.9)	NR	RHIR, LHIR, and OHIR	Postoperative complications	**Age > 65 years was significantly associated with the use of robotic compared to non-robotic surgery (OR 1.01, p = 0.002)**
Pini et al. (2021) [49]	USA	Retrospective cohort study	2017–2019	51 (100)	63.1 (12.7)	NR	RTAPP + suturing and fixation of the transversalis fascia to the Cooper ligament	Seroma formation, recurrence, chronic pain, LOS	No conclusion based on age

*USA* United states of America

Type of procedure: *R* Robotic/ *L* laparoscopic / *O* Open, *IHR* Inguinal Hernia Repair, *TAPP* Transabdominal Preperitoneal, *TEP* total extraperitoneal

Outcomes: *OT* Operative Time, *LOS* Length of stay, *VAS* Visual Analog scale of pain, *SSI* Surgical site infection, *NSAID* Non-steroidal anti-inflammatory drugs

Reported conclusions based on age are in bold

### Age-based conclusion

There was no age-based conclusion in 73% (n = 29) of the studies. Among those that offered age-associated conclusions (n = 9), three studies reported that an age greater than 70 years is a risk factor for postoperative complications, with the risk proportionally increasing each year of life. In four studies, age differences were reported based on the inguinal hernia repair group (open, laparoscopic, or robotic), but the predominance varied according to the study. Two studies concluded that RIHR is feasible and safe in patients aged 80 years and older.

## Discussion

The aim of the study was to provide evidence that clarifies the effect of patient’s age on RIHR clinical outcomes, both from the point of view of a single institution and single surgeon experience and from the perspective of a comprehensive systematic review without precedent in the literature. We studied 37 patients who underwent such procedure and found that there were no differences in surgical outcomes and postoperative complication rates between patients under or over 70 years of age. Simultaneously, a systematic review was conducted on the highest-available evidence from studies associated with RIHR to determine their conclusions and observations based on age groups. The results from both approaches were helpful for the development of the following discussion. As mentioned before, this is the first systematic review dedicated to the study of transabdominal robotic inguinal hernia repair in patients under and over 70 as a relevant cutoff age to consider complications.

Our study’s findings indicated no significant differences in surgical outcomes and postoperative complication rates between patients based on sex or age under or over 70 years, aligning with the existing literature. For example, Tam et al. (2019) conducted a study on 922 patients who underwent RIHR were predominantly comprised of white men between 51 and 70 years old with similar conclusions [38]. Similarly, Maas et al. (2021) found no differences based on sex or age in their retrospective cohort study of 43 participants who were subjected to RIHR [44]. Notably, our analysis of BMI differences showed younger patients having a slightly higher BMI on average, though not statistically significant, contributing to the ongoing discussion about the role of patient physical characteristics on surgical outcomes. This is particularly relevant given the increasing prevalence of obesity worldwide and its potential impact on surgical risks and recovery [50].

The American Society of Anesthesiologists Physical Status Classification System is a widely used risk stratification tool for preoperative patients. Our study revealed that ASA Class II was the most common classification in our cohort. Pereira et al. (2022) reported in their study of 312 patients undergoing RIHR that ASA Class II was assigned to 58% of patients, and Class III was assigned to 29% [36]. In our analysis we did not find a direct correlation between ASA Class and RIHR outcomes, corroborated with Pereira et al. (2022) in their retrospective analysis, which demonstrated no association with ASA Class and complications in the first month or increased OR conversion to an open approach, either.

Our systematic review and analysis underscores the importance of considering comorbidities and risk factors in the context of RIHR. Hypertension, the most common comorbidity in our study, did not adversely affect surgical outcomes, which is consistent with other studies such as Maas et al. (2021), who reported that RIHR is effective and safe despite various comorbidities present in their cohort, suggesting that well-managed comorbid conditions may not significantly impact the success of minimally invasive surgeries like RIHR [44, 51, 52].

Our results did not find a significant difference in mean operating time (OT) or length of stay (LOS) based on the age group of patients undergoing RIHR. Although both measurements were slightly higher in the cohort of patients aged > 70 years. None of the studies included in the systematic review mentioned any differences in OT or LOS based on the age of the patients.

Different studies on RIHR mention OTs similar to those reported in our study. For instance, the study by Pini et al. in 2021 reported the outcomes of patients undergoing RIHR with a TAPP approach, with a mean age of 63.1 years and an OT of 92.0 ± 23.5 min, which is comparable to our study’s findings with a mean age of 64.8 years and an average OT of 97.6 min [49]. On the other hand, lower OTs were observed in groups of patients undergoing the same procedure but with a lower mean age. For example, Cuccurullo et al. in 2020 reported on patients with a mean age of 48.6 years and an OT of 54.8 min [43], and Arcerito et al. in 2016 reported a mean age of 56 years and an OT of 52 min [39]. However, there are variations to this observation across the different studies analyzed. In other surgical procedures such as laparoscopic cholecystectomy or pancreatic resection, no differences in OT have been reported based on age group (< 70 vs > 70 years) [53–55].

In terms of hospitalization time, no clear trend was observed concerning age among the different studies reporting RIHR with a TAPP approach. Studies such as Kudsi et al. in 2021 reported results for patients over 80 years old with an average LOS of 0.5 days [13]. Pini et al., with a similar average age to ours, reported LOS of 1.3 days [49], while Kakiashvili et al. reported a LOS of 24 days in a group of patients with an average age of 56 years [10]. Although no trend was observed among patients undergoing RIHR, studies like Lin et al. in their systematic review and meta-analysis report longer LOS in older patients undergoing cardiac, oncological, general, vascular, and hip fracture surgeries [56–58].

Regarding the incidence of surgical complications, the most common was found to be the formation of a seroma in 32.4% of patients undergoing RIHR with the TAPP approach, similar to that reported by Mass et al. in 2021, where seroma formation (25%) and groin hematoma (2.3%) were the main complications [44]. Similar complications have been reported, including urinary retention, recurrence, and chronic pain, albeit to a lesser extent in the studies by Cuccurullo et al. [43] and Tam et al. [38].

Only one of the examined articles mentioned an age-based variation in the occurrence of complications. Growing older was linked to problems at 30 days, per Pereita et al.’s 2022 data (adjusted odds ratio [aOR] 1.02, 95% confidence interval [CI] 1.01–1.04). It’s interesting to note that in a cohort of 312 patients having RIHR, the probability of a 30-day complication increased by 2% (OR = 1.02, 95% CI 1.001–1.042) with each additional year [36]. Likewise, our research revealed that patients over 70 years old had a greater frequency of all problems (< 0.001).

The conclusions of the present study should be interpreted within the context of certain limitations. The small sample size may have limited the possibility of finding statistically significant differences among the evaluated variables. However, within the field of RIHR, very few studies have addressed differences in clinical outcomes based on patients’ age, as conducted in this study, such as selection bias, confounding variables, and the inability to establish causality. While the systematic review (SR) provided valuable insights, several limitations related to it must be acknowledged. Firstly, the inclusion of observational retrospective studies introduces inherent biases and limitations associated with this study design. Additionally, the quality and heterogeneity of the included observational studies may vary, potentially impacting the robustness and generalizability of the findings.

This study provides valuable insights into RHIR and underscores the need for further research to address the definitive impact of age in the surgical outcomes of this technique. Therefore, future durations for research should include higher-quality studies such as randomized controlled trials or prospective multicentric international clinical trials with robotic inguinal hernia repair in patient populations typically considered to be at higher risk for complications with an open surgical approach.

## Conclusion

In our single center, single surgeon experience, there were no statistically significant changes in operating times, length of hospital stay, comorbidities, risk factors, or ASA classification between patients in the two age groups (under 70 vs. 70 and older). In contrast to younger patients, patients over 70 had a higher frequency of complications. While this conclusion is supported by the available research, it is also proposed that, for both younger and older persons, the robotic technique provides a safe and minimally invasive substitute for open or laparoscopic procedures. More extensive prospective investigations and randomized clinical trials are required to evaluate the effect of aging on complications following surgery.

## Data Availability

The corresponding author can provide the data that supported the study’s conclusions upon request. The information cannot be made available to the public or included in data repositories due to privacy or confidentiality issues. Nonetheless, we are dedicated to upholding the accuracy, openness, and repeatability of our research, and we are ready to grant interested parties access to the data upon request.
